# Serial Fetal Heart Rate Monitoring During Electroconvulsive Therapy in Late Pregnancy: A Case Report of Transition From Succinylcholine to Rocuronium Reversed With Neostigmine

**DOI:** 10.1002/npr2.70159

**Published:** 2026-09-02

**Authors:** Katsuichiro Shimizu, Hiroyuki Muraoka, Tomoe Fujita, Kyoko Hattori, Taiyo Nishikawa, Hana Uchitsubo, Daigo Ochiai, Shunsuke Hyuga, Ken Inada

**Affiliations:** ^1^ Department of Psychiatry Kitasato University School of Medicine Sagamihara Japan; ^2^ Division of Obstetric Anesthesia, Center for Perinatal Care, Child Health and Development Kitasato University Hospital Sagamihara Japan; ^3^ Department of Anesthesiology Kitasato University School of Medicine Sagamihara Japan; ^4^ Department of Obstetrics and Gynecology Kitasato University School of Medicine Sagamihara Japan

**Keywords:** electroconvulsive therapy, fetal monitoring, neostigmine, pregnancy, rocuronium

## Abstract

Electroconvulsive therapy (ECT) is considered relatively safe and effective for severe psychiatric disorders during pregnancy, but evidence regarding neuromuscular blockers for ECT in pregnancy remains limited. We report a 30‐year‐old primigravida admitted at 30 weeks of gestation with severe major depressive disorder and persistent suicidal ideation despite pharmacotherapy. ECT was initiated at 32 weeks and 2 days of gestation. The first five sessions used succinylcholine. Owing to a nationwide shortage in Japan, the muscle relaxant was switched to rocuronium from the sixth session, with neostigmine and atropine used for reversal during the two antepartum rocuronium sessions. Serial cardiotocographic monitoring before and after each of the seven antepartum ECT sessions showed a transient decrease in fetal heart rate variability after ECT in all sessions and a transient increase in fetal heart rate baseline in five of the seven sessions (Sessions 1, 2, 3, 5, and 7), while fetal well‐being was maintained throughout. Spontaneous vaginal delivery occurred at 37 weeks and 0 days, five days after the seventh ECT session. The infant developed respiratory failure shortly after birth and required ventilatory support for two days, but subsequently recovered without complications. Postpartum ECT was resumed for five additional sessions using rocuronium reversed with sugammadex, and the patient achieved clinical remission. This case suggests the feasibility of rocuronium with neostigmine reversal as an alternative to succinylcholine for ECT during late pregnancy when succinylcholine is unavailable, provided that multidisciplinary management and fetal monitoring are available.

## Abstract 2

In a pregnant patient with severe major depressive disorder, succinylcholine was replaced by rocuronium with neostigmine–atropine reversal during late‐pregnancy ECT because of a drug shortage. Serial fetal monitoring showed only transient changes, supporting feasibility under multidisciplinary management.

## Graphical abstract

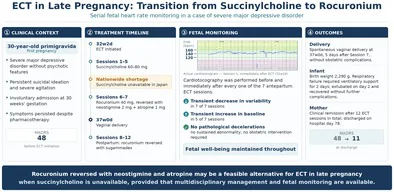

## 1 Background

Electroconvulsive therapy (ECT) is considered a relatively safe and effective treatment for severe psychiatric disorders during pregnancy, particularly when rapid symptom control is required or pharmacotherapy is ineffective or contraindicated [1, 2]. The estimated prevalence of depression during pregnancy is approximately 12% [3], and treatment decisions require careful balancing of risks to both the mother and fetus.

Anesthetic management for ECT typically involves intravenous induction followed by a neuromuscular blocker. Succinylcholine has traditionally been the standard neuromuscular blocker for ECT because of its rapid onset and short duration [4]. However, recent shortages, including a nationwide supply constraint in Japan, have required alternative strategies [5]. Rocuronium is a potential substitute but requires reversal with neostigmine or sugammadex, and empirical data on its use for ECT during pregnancy remain limited [6, 7, 8, 9, 10].

To our knowledge, no detailed report has described an in‐pregnancy transition from succinylcholine to rocuronium within the same patient, with cardiotocographic monitoring spanning both treatment phases. We report a case of severe major depressive disorder with persistent suicidal ideation in a primigravid woman who underwent 12 ECT sessions spanning late pregnancy, full‐term delivery, and the early postpartum period.

## 2 Case Presentation

A 30‐year‐old primigravid Japanese homemaker with no significant personal medical or psychiatric history, and a family history of bipolar disorder in her mother, developed psychiatric symptoms during her first pregnancy. She experienced severe hyperemesis gravidarum, leading to poor oral intake, weight loss, and three admissions to a local obstetrics clinic. During her third admission at 15 weeks of gestation, she developed severe anxiety, agitation, suicidal ideation, and wandering behavior. At 18 weeks of gestation, she was referred to our hospital. Quetiapine was initiated primarily for severe anxiety, agitation, and insomnia, with initial improvement. Bipolar‐spectrum depression was considered because of the maternal family history and marked agitation, but no manic or hypomanic episode had been identified. Around 30 weeks of gestation, however, depressive mood, anxiety, agitation, and suicidal ideation re‐emerged, prompting involuntary admission to our department.

On admission, she exhibited depressed mood, anhedonia, anorexia, insomnia, agitation, indecisiveness, and persistent suicidal ideation. No hallucinations or delusions were observed. In the absence of any history of manic or hypomanic episodes, she was diagnosed with major depressive disorder, severe, without psychotic features, according to the Diagnostic and Statistical Manual of Mental Disorders, Fifth Edition. Duloxetine 20 mg/day was initiated, but because her symptoms did not improve, ECT was started on hospital day 14 at 32 weeks and 2 days of gestation. Her Montgomery–Åsberg Depression Rating Scale (MADRS) score was 48 before ECT initiation [11]. Her agitation was severe; she was unable to remain in bed and occasionally engaged in self‐injurious behavior.

**FIGURE 1:**
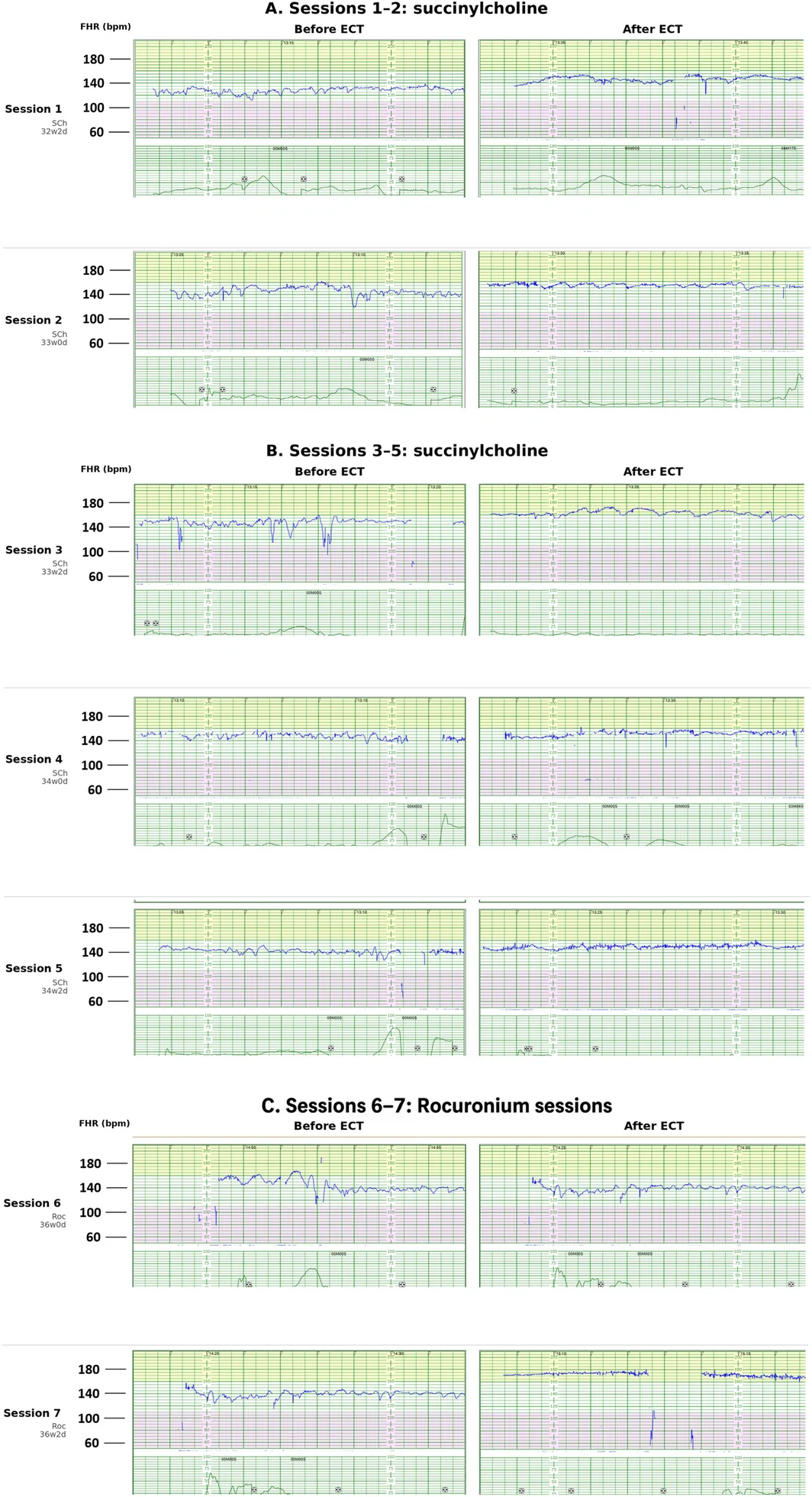
Serial fetal heart rate monitoring before and after each of the seven antepartum electroconvulsive therapy sessions. Panel A shows Sessions 1–2, Panel B shows Sessions 3–5, and Panel C shows Sessions 6–7. Each row represents one electroconvulsive therapy (ECT) session with gestational age. The left and right panels show cardiotocographic tracings obtained immediately before and after ECT stimulation, respectively. In each panel, the upper trace represents fetal heart rate and the lower trace represents uterine activity. For readability, auxiliary fetal heart rate scale labels were added outside the original tracings; these labels are visual aids and do not alter the original clinical recordings. Sessions 1–5 were performed with succinylcholine; from Session 6, the muscle relaxant was rocuronium, reversed with neostigmine and atropine during the two antepartum rocuronium sessions. Transient post‐ECT changes in fetal heart rate variability and baseline were observed, but no findings suggestive of fetal compromise, including pathological decelerations or sustained baseline abnormalities, were detected in any session.

Twelve ECT sessions were performed using a Thymatron System II device (Somatics LLC), bitemporal electrode placement, brief‐pulse stimuli, intravenous propofol, and face‐mask ventilation with 100% oxygen (Table 1). Cardiotocography was performed before and immediately after each of the seven antepartum ECT sessions (Figure 1). After ECT, a transient decrease in fetal heart rate variability was observed in all sessions, and a transient increase in fetal heart rate baseline was observed in five of the seven sessions (Sessions 1, 2, 3, 5, and 7). No sustained maternal instability or obstetric intervention was required (Table 1).

**TABLE 1:** Summary of electroconvulsive therapy sessions, anesthetic management, seizure metrics, maternal responses, and fetal monitoring findings.

Session	GA	Neuromuscular management	Propofol (mg)	Stimulus intensity (%)	Seizure metrics EEG duration (s); PSI (%)	Maternal response BP (mmHg); HR (beats/min); SpO_2_ (%)	Fetal response
A. Antepartum ECT sessions and delivery							
1	32w2d	SCh 60 mg	100–120	15	EEG 97 PSI N/A Adequate	BP 125/88 → 170/112 HR 75 → 110 SpO_2_ nadir 94	Transient ↓ variability Transient ↑ baseline No fetal compromise
2 (1st)	33w0d	SCh 60 mg	100–120	15	No EEG seizure PSI N/A Failed seizure induction	BP 137/75 → 187/110^a^ HR 83 → 90^a^ SpO_2_ nadir 92^a^	Transient ↓ variability^a^ Transient ↑ baseline^a^ No fetal compromise
2 (2nd)	33w0d	No additional dose	—	25	EEG 72 PSI N/A Adequate after re‐stimulation	Same session^a^	Same session^a^
3	33w2d	SCh 80 mg	100–120	25	EEG 37 PSI 91.6 Adequate	BP 120/63 → 175/112 HR 99 → 99 SpO_2_ nadir 92	Transient ↓ variability Transient ↑ baseline No fetal compromise
4	34w0d	SCh 80 mg	100–120	25	EEG 23 PSI 45.3 Adequate	BP 110/70 → 163/107 HR 100 → 125 SpO_2_ nadir 96	Transient ↓ variability No baseline increase No fetal compromise
5	34w2d	SCh 80 mg	100–120	25	EEG 22 PSI N/A Adequate	BP 125/85 → 183/113 HR 90 → 110 SpO_2_ nadir 97	Transient ↓ variability Transient ↑ baseline No fetal compromise
6	36w0d	Roc 40 mg → Neo 2 mg + atropine 1 mg	220	35	EEG 35 PSI N/A Adequate	BP 110/70 → 200/112 HR 60 → 150 SpO_2_ nadir 97	Transient ↓ variability No baseline increase No fetal compromise
7 (1st)	36w2d	Roc 40 mg → Neo 2 mg + atropine 1 mg	120	35	EEG 6 PSI N/A Inadequate brief seizure	BP 87/56 → > 200/110^a^ HR 63 → 150^a^ SpO_2_ nadir 92^a^	Transient ↓ variability^a^ Transient ↑ baseline^a^ No fetal compromise
7 (2nd)	36w2d	No additional dose	—	50	EEG 15 PSI N/A Adequate after re‐stimulation	Same session^a^	Same session^a^
Delivery	37w0d	—	—	—	—	—	Birth weight 2290 g Apgar scores 3/6/8

Session	Postpartum day	Rocuronium (mg)	Sugammadex (mg)	Propofol (mg)	Stimulus intensity (%)	Seizure outcome
B. Postpartum ECT sessions						
8	PPD 7	50	500	120	75	Adequate
9	PPD 9	50	500	120	75	Adequate
10	PPD 21	50	500	120	75	Adequate
11	PPD 23	50	600	120	75	Adequate
12	PPD 28	60	600	150	100	Adequate

*Note:* Maternal blood pressure and heart rate are shown as the closest available pre‐stimulation values and the peak post‐stimulation values. SpO_2_ represents the lowest value recorded during the session. Maternal vital‐sign values were retrospectively extracted from anesthesia record graphs and should be interpreted as approximate values. Motor seizure duration was not systematically recorded because electromyographic monitoring was not used during ECT sessions. PSI was automatically calculated by the ECT device when available; N/A indicates that no calculable value was output by the device or retained in the clinical record. No manual calculation of PSI was performed. An adequate seizure was defined as an ECT‐induced seizure judged by the treating team to be therapeutically sufficient based on EEG seizure duration, EEG seizure morphology, PSI when available, and clinical observation. In Session 2, the initial stimulation failed to induce an EEG seizure. In Session 7, the initial stimulation induced a brief 6‐s EEG seizure that was judged inadequate. In both sessions, re‐stimulation at a higher intensity resulted in an adequate seizure.

Abbreviations: BP, blood pressure; ECT, electroconvulsive therapy; EEG, electroencephalography; GA, gestational age; HR, heart rate; N/A, not available; Neo, neostigmine; PPD, postpartum day; PSI, postictal suppression index; Roc, rocuronium; SCh, succinylcholine; SpO_2_, peripheral oxygen saturation.

^a^ For sessions requiring re‐stimulation, maternal vital signs and fetal monitoring findings represent the overall ECT session rather than each individual stimulation. For Session 7, the pre‐stimulation blood pressure value could not be assigned definitively to the first or second stimulation.

Details of each ECT session are shown in Table 1. Sessions 1–5 were performed with succinylcholine. Because succinylcholine became unavailable, the muscle relaxant was switched to rocuronium 40 mg from session 6, at 36 weeks and 0 days of gestation. During the two antepartum rocuronium sessions, neuromuscular blockade was reversed with neostigmine 2.0 mg and atropine 1.0 mg [12]. Recovery from neuromuscular blockade was assessed using train‐of‐four (TOF) monitoring, and a TOF ratio of ≥ 0.9 was confirmed before transfer from the treatment room in both sessions. In Session 6, propofol 120 mg was followed by 20‐, 20‐, 30‐, and 30‐mg supplemental doses (total 220 mg); their timing and indication could not be determined retrospectively. Session 6 produced an adequate seizure. At session 7, the first stimulation produced a brief 6‐s EEG seizure that was judged inadequate, and an adequate seizure was obtained after re‐stimulation at a higher intensity without additional anesthetic or muscle relaxant.

Five days after Session 7, spontaneous vaginal delivery without obstetric complications occurred at 37 weeks and 0 days; her MADRS score was 36. Planned elective induction was pre‐empted by spontaneous labor. The infant weighed 2290 g, which was classified as low birth weight despite delivery at 37 weeks of gestation, with Apgar scores of 3, 6, and 8 at 1, 5, and 10 min, respectively. Antenatal fetal growth was monitored by the obstetric team, and fetal growth restriction was not suspected before delivery. Umbilical cord blood gas analysis showed pH 7.241, base excess −2.1, and lactate 28.2 mg/dL, without severe metabolic acidosis. Pediatric diagnoses included transient tachypnea of the newborn, suspected neonatal withdrawal syndrome, neonatal asphyxia based on low Apgar scores, and neonatal hypoglycemia. Chest radiography was unremarkable, with no intrapartum fetal distress, congenital anomaly, or clinical evidence of infection. The infant developed respiratory failure shortly after birth and required endotracheal intubation and ventilatory support in the neonatal intensive care unit. The infant was extubated on day 2, weaned from oxygen on day 3, and discharged on day 19.

Immediately postpartum, the patient appeared calm. However, on the following day she stated, “I want to die now that the delivery is over,” and her suicidal ideation intensified. Behavioral disturbance recurred, including wrapping cords around her neck, and severe agitation required seclusion. Her MADRS score re‐escalated to 45. Duloxetine, which had been titrated to 60 mg/day, was discontinued and replaced with mirtazapine. ECT was resumed postpartum for five additional sessions using rocuronium with sugammadex reversal (Table 1). The patient initially declined contact with her infant, but after session 9 she expressed a wish to see the infant and began visiting the neonatal ward. Her agitation and suicidal ideation resolved, and depressive symptoms gradually improved. At discharge, mirtazapine 45 mg/day was maintained and lithium carbonate 400 mg/day was added for post‐ECT relapse prevention, considering recurrence risk, severe suicidality, and possible bipolar‐spectrum vulnerability. She was discharged on hospital day 78 in clinical remission, with a MADRS score of 11.

Written informed consent was obtained from the patient for publication of this case report and any accompanying clinical data and images.

## 3 Discussion and Conclusion

This case describes ECT in late pregnancy and early postpartum, including an unplanned switch from succinylcholine to rocuronium and serial antepartum cardiotocography. Recent systematic review evidence indicates that the literature on ECT during pregnancy remains based largely on case reports and case series [13].

Succinylcholine remains the conventional neuromuscular blocker for ECT because of its rapid onset and short duration, but its use during pregnancy is based mainly on accumulated clinical experience [4]. Rocuronium crosses the placenta only in small amounts and has not been associated with adverse fetal outcomes during general anesthesia for cesarean delivery [10], but published experience with its use for ECT in pregnant women remains limited.

The choice of reversal agent was an important feature of this case. Sugammadex provides rapid reversal of rocuronium, but its use during pregnancy remains cautious because of limited clinical safety data and theoretical concerns regarding progesterone activity [7, 8]. We therefore used neostigmine with atropine for antepartum reversal and reserved sugammadex for postpartum sessions. Because neostigmine crosses the placenta and may cause fetal bradycardia when muscarinic effects are unopposed, atropine was co‐administered [9].

Although transient changes in fetal heart rate variability and baseline were observed after ECT, no pathological decelerations, sustained baseline abnormalities, or findings requiring obstetric intervention were detected. Interpretation of these findings requires caution because the switch to rocuronium coincided with an increase in stimulus intensity, the total propofol dose was higher in Session 6 because supplemental doses were administered, and seizure characteristics and maternal cardiovascular responses differed across sessions. Accordingly, the fetal heart rate changes cannot be attributed specifically to rocuronium or neostigmine. This case supports the feasibility of this approach under multidisciplinary monitoring rather than comparative safety between neuromuscular blocker strategies.

The initial stimulation in Session 2 failed to induce an EEG seizure, whereas that in Session 7 produced a brief 6‐s EEG seizure judged inadequate. In both sessions, an adequate seizure was obtained after re ‐ stimulation at higher intensity.

ECT anesthesia during pregnancy shares concerns with general anesthesia, including maternal oxygenation and hemodynamics, airway and aspiration risk, uteroplacental perfusion, and fetal monitoring [14]. Unlike surgical anesthesia, ECT is brief and lacks surgical trauma but involves electrical stimulation, an induced seizure, transient autonomic responses, and repeated exposures. Thus, data on rocuronium and reversal agents from general anesthesia or cesarean delivery are informative but not directly generalizable to ECT.

Bipolar‐spectrum depression remained a differential diagnosis because of family history, agitation, and treatment resistance, but no lifetime or in‐hospital mania or hypomania was identified; therefore, major depressive disorder was retained. Quetiapine was used primarily for anxiety, agitation, and insomnia, while longitudinal diagnostic reassessment remains warranted. Lithium was added for post‐ECT relapse prevention, given recurrence and suicide risk and possible bipolar‐spectrum vulnerability; lithium‐containing continuation pharmacotherapy can reduce relapse after successful ECT for major depression [15].

The neonatal respiratory failure was an important adverse perinatal event. Differential diagnoses included transient tachypnea, respiratory distress syndrome, poor neonatal adaptation or withdrawal, perinatal asphyxia, infection, congenital anomaly, and ECT‐ or anesthesia‐related compromise. Pediatric diagnoses included transient tachypnea of the newborn and suspected neonatal withdrawal syndrome. Although neonatal asphyxia was also recorded based on low Apgar scores, cord blood gas analysis showed no severe metabolic acidosis, and there was no intrapartum fetal distress, congenital anomaly, or clinical evidence of infection. Rapid improvement, with extubation on day 2 and oxygen discontinuation on day 3, supported transient neonatal respiratory adaptation. Third‐trimester serotonergic antidepressant exposure has been associated with poor neonatal adaptation, although individual causality cannot be established [16]. Transient tachypnea with possible poor neonatal adaptation or withdrawal was considered most plausible. A direct relationship with ECT, rocuronium, or neostigmine was considered unlikely but cannot be excluded in a single case.

Several limitations should be acknowledged. First, this is a single case, and conclusions about safety must be interpreted cautiously. Second, long‐term neurodevelopmental follow‐up of the infant beyond the perinatal period was not undertaken. Third, only five sessions used succinylcholine and only two antepartum sessions used rocuronium; sugammadex was not used during pregnancy. Fourth, motor seizure duration was not systematically recorded because electromyographic monitoring was not used, and PSI was unavailable for some sessions. Maternal vital signs were retrospectively extracted from anesthesia record graphs and are approximate. Finally, the present case occurred under conditions in which succinylcholine was unavailable, and the relative benefit–risk balance of routinely choosing rocuronium when succinylcholine is accessible cannot be inferred.

In conclusion, this case suggests that rocuronium reversed with neostigmine may be a feasible alternative to succinylcholine for ECT in late pregnancy when succinylcholine is unavailable, provided that multidisciplinary management and fetal monitoring are available. Further case accumulation is needed to establish standardized protocols for neuromuscular blocker and reversal agent selection during ECT in pregnancy.

## Author Contributions

K.S. provided psychiatric treatment and drafted the manuscript. H.M. supervised psychiatric management and substantively revised the manuscript. T.F. and S.H. provided anesthetic management during ECT, including neuromuscular blockade and reversal management. T.N. contributed to psychiatric management. K.H., H.U., and D.O. provided obstetric care, including labor and delivery management and fetal monitoring. K.I. provided overall supervision and final review. All authors read and approved the final manuscript.

## Funding

The authors have nothing to report.

## Ethics Statement

This single‐patient case report did not require formal institutional review board approval according to the policy of our institution. Written informed consent for publication was obtained from the patient.

## Consent

Written informed consent was obtained from the patient for the publication of this case report, including any accompanying clinical data and images.

## Conflicts of Interest

The authors declare no conflicts of interest.

## Data Availability

The individual‐level clinical data underlying this case report cannot be publicly archived or shared because they contain potentially identifiable patient information and are restricted by the scope of the patient's consent. All relevant de‐identified clinical information necessary to understand the case is included in this article.
